# Supplementary material for: Sex differences in the incidence, mortality, and fatality of COVID-19 in Peru
Source: PLoS One. 2021 Jun 14;16(6):e0253193. doi: 10.1371/journal.pone.0253193 (PMC8202928; doi:10.1371/journal.pone.0253193)
Supplement: Attachment — Submitted filename: Response-to-review_PONE-D-21-06294.docx [file pone.0253193.s001.docx]

**Response-to-reviewers: Manuscript** PONE-D-21-06294

We thank the Reviewers for their comments and constructive criticism, we believe that the quality of our manuscript has been significantly improved. We have revised our paper in a point-by-point manner. Modifications are in yellow text.

Reviewer #1: This study highlights the COVID-19 incidence and mortality among the Peruvian population. The authors show that the incidence as well as mortality rate is high for males compared to females. This is a general trend in most of the countries. This study does not highlight new findings; however, as the authors have indicated, this study could be important for monitoring and introducing infection control measures in Peru. The manuscript is well written and the authors have presented the data comprehensively. Hence, this manuscript can be accepted in the current form.

Response: Thank you for your comment.

Reviewer #2: The present study shows higher COVID-19 incidence and related mortality among men than women in Peru. In addition to showing that men are at greater risk of COVID-19 death, the authors also shows that fatality rates increased with age, and that were mainly predominant in men with 50 years of age or older. The present study lacks in novelty, since these findings are only consistent with a very large number of published reports from different European countries, from different Asian countries, including China, and from the USA. The only advantage that I see from this study is the geographical area of assessment, beeing a research study performed in Peru, since COVID-19 reports from South America are still too few compared to the other continents.

The authors need to add a paragraph in the Discussion on the potential mechanisms involved in the higher COVID-19 incidence and related mortality among men than women. You can also cite and use as a reference the following very recent publication: Gender differences in the battle against COVID-19: Impact of genetics, comorbidities, inflammation and lifestyle on differences in outcomes. Int J Clin Pract. 2021 Feb;75(2):e13666.

Response: Thank you for your comment. We have included a paragraph in the Discussion on the potential mechanisms involved in the higher COVID-19 incidence and related mortality among men than women (lines, 228-247). We have also cited and use as a reference the publication: Gender differences in the battle against COVID-19: Impact of genetics, comorbidities, inflammation and lifestyle on differences in outcomes. Int J Clin Pract. 2021 Feb;75(2):e13666.

Reviewer #3: The author report higher incidence, mortality and fatality rates among men than among women from Peru. Men are at greater risk of COVID-19 death. The mortality rates increased with age, and were most predominant in men 50 years of age or older.
The manuscript is well written and technically sound. However, for a global audience the manuscript lacks novelty. The gender and age as risk factors for COVID-19 are already well described.

Response: Thank you for your comment. While the gender and age as risk factors for COVID-19 are already well described in different European countries, including China, and from the USA, COVID-19 reports in South America and Peru are still too few. Therefore, actions aimed at improving the surveillance and prevention of infection in the population, are necessary in a country where the capacity for hospital response and intensive care is insufficient. We also understand that novelty is not a criterion for acceptance at PLOS ONE.
